# Clinicians’ Decision-Making Regarding Telehealth Services: Focus Group Study in Pediatric Allied Health

**DOI:** 10.2196/46300

**Published:** 2024-06-07

**Authors:** Donna Claire Thomas, Eva Frances Litherland, Sarah Masso, Gianina Raymundo, Melanie Keep

**Affiliations:** 1 Sydney School of Health Sciences Faculty of Medicine and Health The University of Sydney Camperdown Australia; 2 Integrated and Community Health Western Sydney Local Health District Blacktown Australia

**Keywords:** telehealth, pediatric, allied health, focus group, decision-making, community-based, counseling, speech pathology, occupational therapy, clinical services

## Abstract

**Background:**

Many allied health services now provide both telehealth and in-person services following a rapid integration of telehealth as a response to the COVID-19 pandemic. However, little is known about how decisions are made about which clinical appointments to provide via telehealth versus in person.

**Objective:**

The aim of this study is to explore clinicians’ decision-making when contemplating telehealth for their clients, including the factors they consider and how they weigh up these different factors, and the clinicians’ perceptions of telehealth utility beyond COVID-19 lockdowns.

**Methods:**

We used reflexive thematic analysis with data collected from focus groups with 16 pediatric community–based allied health clinicians from the disciplines of speech-language pathology, occupational therapy, social work, psychology, and counseling.

**Results:**

The findings indicated that decision-making was complex with interactions across 4 broad categories: technology, clients and families, clinical services, and clinicians. Three themes described their perceptions of telehealth use beyond COVID-19 lockdowns: “flexible telehealth use,” “telehealth can be superior to in-person therapy,” and “fear that in-person services may be replaced.”

**Conclusions:**

The findings highlight the complexity of decision-making in a community-allied health setting and the challenges experienced by clinicians when reconciling empirical evidence with their own clinical experience.

## Introduction

### Background

Telehealth refers to the delivery of health care, including assessment and diagnosis, treatment, monitoring, and information exchange, by a health care provider through the use of information and communication technologies [1]. Despite clear evidence that telehealth is effective [2-4] and has high levels of consumer acceptance [5], prior to the COVID-19 pandemic, there was limited evidence of ongoing and widespread adoption of telehealth generally and in community and allied health settings specifically [6].

However, from 2020 onward, the COVID-19 pandemic changed the landscape of telehealth use. For health services providing nonurgent care, COVID-19–related public health restrictions resulted in an exponential growth in telehealth use [7-10]. Following this expansion in telehealth use, many clinicians have called for an ongoing role for telehealth in a hybrid or blended capacity, with a combination of telehealth and in-person sessions [11,12], and a better understanding of the complexity of the clinician, service, consumer, and system factors involved in telehealth sustainability [13].

There is currently limited information about how clinicians decide which clinical sessions should be offered via telehealth within a blended telehealth and in-person service delivery model. Although there is a general awareness that clinicians consider client factors when making clinical decisions, as reflected in numerous implementation science frameworks (eg, the Nonadoption, Abandonment, Scale-Up, Spread, and Sustainability [NASSS]) [7,14], there are limited details about how these factors should be considered. Cook et al [15] noted that, when working with adult clients, allied health clinicians’ decisions are often driven by the clinicians’ assumptions and preconceived beliefs. The existing guidelines for telehealth use in allied health only indicate that eligibility should be considered on a case-by-case basis [16,17], informed by professional judgment [17,18], and in consideration of individual patients’ characteristics [19].

Like all clinical decisions, the decision about treatment modality—telehealth or in person—involves choosing between alternatives [20]. Clinical decision-making, in general, is complex and requires gathering and evaluating data prior to making a decision [14]. There are 2 primary methods for clinical decision-making [21]: the information-processing model and the intuitive-humanist model [22]. Clinical decisions made using an information-processing model require deliberate and analytical processing [21]. This approach is founded on logic and uses a series of hypotheses and deductions, where clinicians actively seek and evaluate information [22]. The information-processing model of decision-making is comprehensive but time-consuming [21]. The alternative method of decision-making is the intuitive-humanist model. Intuitive decision-making relies heavily on knowledge gained from experience, making it a quick and low effort for clinicians [21]. With experience, a clinician observes patterns and themes, quickly differentiating relevant from irrelevant information and intuitively knowing which decision to make. However, if the clinician relies on incorrect information, their pattern matching may be incorrect [23], leading to gaps between evidence and practice [21], particularly if the clinician does not use information processing as a check mechanism [21].

Alongside specific patient and client factors—and the relative suitability of telehealth for a specific client group—clinicians are routinely expected to adhere to principles of evidence-based practice (EBP). One model of EBP, described by Dollaghan [24] (called E_3_BP), describes three different sources of evidence that clinicians should use in decision-making: (1) external evidence from empirical investigations, (2) internal evidence from clinicians’ own experience, and (3) the preferences of an informed client or caregiver. EBP models outline the different—sometimes competing—sources of evidence that need to be considered when selecting telehealth or in-person service.

The transition of a service to a telehealth model, particularly a rapid transition to telehealth as seen during the COVID-19 pandemic, may place strain on clinicians’ ability to engage with EBP. In different areas of allied health, telehealth assessment has been found to be valid and reliable [25,26]. Likewise, telehealth is effective for many allied health interventions [27-30]. However, the rapid transition to telehealth in recent years has left some clinicians scrambling to implement known assessment and intervention procedures, based on clinical expertise, in new technology-driven contexts. In this way, clinicians rated some areas of clinical practice as considerably more challenging to conduct via telehealth than others [31]. For example, evaluations of swallowing and feeding, as well as speech sound production, were the most difficult for speech-language pathologists (SPs) surveyed during the COVID-19 pandemic [31], and the provision of musculoskeletal intervention was difficult for physiotherapists [32]. The pandemic forced many services to adopt telehealth as a service delivery option despite these challenges. Services that experienced a rapid transition to telehealth are now faced with the decision as to whether ongoing telehealth services are suitable for their clients—and if so, which clients—beyond the context of the pandemic [10].

This study aimed to understand the factors considered by pediatric allied health clinicians when choosing to recommend telehealth to a client and how these factors are balanced and considered. Specifically, the research questions were as follows: (1) How do clinicians decide whether to recommend telehealth for their clients and which factors do they consider using when making these decisions? and (2) What are clinicians’ perceptions of telehealth utility outside of COVID-19 lockdowns?

### Study Context

This study was conducted within the Child and Family Allied Health Services (CFAHS) located in a local health district in metropolitan Sydney, New South Wales (NSW), Australia. The local health district serves an ethnically and culturally diverse population, with 46.8% of residents being born overseas (compared to 29.7% of the total NSW population), and 50.3% speaking a language other than English at home (compared to 26.9% in NSW). In addition, the health service covers a major area of settlement for refugees in NSW [33]. There is a range of socioeconomic advantages in the district, with wealth at one end of the spectrum and significant social disadvantage at the other, bringing with it a range of complex health needs and social circumstances. The CFAHS department of the local health district provides government-funded, community-based health care to children and their families across 3 disciplines: speech-language pathology (0-8 years), occupational therapy (0-8 years), and counseling services (0-18 years). These allied health services are provided from 7 community health centers and are integrated with other colocated disciplines such as child and family health nursing (who provide health, hearing, and vision assessments and developmental screening).

Prior to 2020, all CFAHS clinical care was provided in person at the community health centers, with some home visiting and outreach to local preschools and schools. In March 2020, in response to the COVID-19 pandemic and the associated restrictions on in-person nonurgent clinical care, the CFAHS rapidly implemented telehealth, following the guidelines provided by the NSW Health Agency for Clinical Innovation [34]. Staff were trained in using a videoconferencing platform (Pexip) [35] and resources were adapted or secured for web-based use. In-person services gradually resumed from July 2020 onward, with telehealth or in-person services being at the clinician’s discretion from July 2020 until the period of this study (May and June 2021).

## Methods

### Design

This study used qualitative methodology to gain an in-depth understanding of clinicians’ experiences and perceptions of telehealth use, as well as the factors that influence these experiences [36]. Focus groups with allied health clinicians were used to understand the range of perspectives on clinical decision-making regarding telehealth [37]. The focus groups also allowed the researchers to observe the interaction dynamics between allied health professionals in an environment that reflects interactions in clinical settings [37]. The focus groups explored participants’ experiences of (1) making decisions regarding telehealth versus in-person for the clinical care of their clients and (2) providing clinical care via telehealth outside of COVID-19 lockdown periods.

### Ethical Considerations

This study was granted ethics approval from the Sydney Children’s Hospitals Network Human Research Ethics Committee (approval 2021/ETH00219). All participants provided written consent. No compensation was provided to participants. Data was collected during participants' regular working hours. Data was deidentified by using a code.

### Participants

In total, 16 allied health staff (SPs, occupational therapists [OTs], psychologists, social workers, and counselors) from the CFAHS participated in the study. Clinicians were eligible to participate if they were an allied health staff member, employed at the service for at least 1 month from March to July 2020, and provided clinical services via telehealth as part of their work role. The clinicians represented all 7 physical CFAHS center locations, with some staff working across multiple centers. Table 1 summarizes participants profession, role in the service, and years of experience.

All participants provided allied health clinical services to children and families. Four participants were senior clinicians who also provided clinical supervision, workload allocation, and service coordination for a group of 8-14 clinicians within their own profession.

**Table 1:** Focus group participant (n=16) professional and demographic details.

Characteristic and profession				Participant, n (%)
**Allied health discipline**				
	Speech-language pathology		10 (62)	
	Occupational therapy		2 (12)	
	**Counseling team member**		4 (25)	
		Social work	1 (6)	
		Psychology	1 (6)	
		Counseling	2 (12)	
**Role in service**				
	Clinician		12 (75)	
	**Senior clinician**		4 (25)	
		Speech-language pathologist	2 (12)	
		Occupational therapist	1 (6)	
		Counselor	1 (6)	
**Years of practice in health profession**				
	1-5		5 (31)	
	6-10		2 (12)	
	11-15		5 (31)	
	16-20		0 (0)	
	>20		4 (25)	
**Sex**				
	Female		14 (88)	
	Male		2 (12)	

### Recruitment and Data collection

In March 2021, all eligible allied health staff within the CFAHS team (n=60) were invited to participate in a focus group via email. In total, 16 clinicians (including 4 senior clinicians) expressed interest in the project and participated in 1 of 4 focus groups (3 for clinicians and 1 for senior clinicians) between May and June 2021. Each participant attended 1 focus group on 1 occasion. At the time of the focus groups, in NSW, there were few COVID-19 public health restrictions, and telehealth services in CFAHS were offered to clients at the clinician’s discretion.

Focus groups (mean 56.25 min) were conducted via videoconferencing (Zoom; Zoom Video Communications) with 2 facilitators (DCT and MK), both experienced in qualitative research. The focus groups were transcribed verbatim by a third party, Pacific Transcription Services. Transcriptions were reviewed by DCT for accuracy and emailed to participants for verification. Two (12%) of the 16 participants made minor emendations to the transcription to enhance clarity or correct the grammar.

### Data Analysis

The interview transcripts were analyzed using the reflexive thematic analysis process, initially described by Braun and Clarke [38] and in keeping with their later writings [39]. Transcripts were read by the research team multiple times, and the team met to discuss their initial ideas about the data. The interview transcripts were then coded using NVivo12 software (Lumivero) [40], which involved reviewing all transcripts line by line to understand the underlying ideas and attitudes that were conveyed. These lines or responses were then categorized (coded) into 1 or multiple codes. The initial coding process and secondary coding were reviewed by the research team fortnightly, and the codes changed over time. The research team then met to collate the codes together, to form and refine the overarching themes [39].

### Researcher Reflexivity

The research team has clinical and academic backgrounds in health and psychology and is familiar with the local health district where this research takes place. This means that they were able to draw on similar experiences to their participants to inform their research process [41]. In particular, their reflexivity may have influenced both data collection and data analysis [41]. DCT is a SP and lecturer who provides clinical services and clinical supervision via telehealth, and MK has a psychology degree and lectures and conducts research in the field of eHealth but is not a practicing clinician. The health background of the focus group facilitators could have influenced their rapport with the participants, the direction of the discussion, and the depth of responses received during the focus group discussions [41]. The other team members involved in data analysis were EL, a Community and Integrated Health Service manager at Western Sydney Local Health District at the time of writing, and GR, who was conducting doctoral research into health service experiences among young adults from migrant backgrounds. Their understanding of the context of the participants’ professional practice may have also influenced the group discussions about coding, the responses that were coded, and the way these codes were collated into final themes [41,42].

## Results

### Overview

In response to the research question “Which factors do you consider when deciding whether to recommend telehealth?” the participants noted there were “so many variables” [counselor 3] and that “a one rule fits all model” [SP3] does not work because “factors like age, family engagement, technology...all play into how the family engage with telehealth” [SP3]. There were, however, 4 broad categories that clinicians considered: technology, client and family aspects, clinician-related aspects, and clinical presentation. Table 2 consists of a list of the factors within each category and the manner in which the category influenced decision-making. Example quotes for each of the factors are available in Multimedia Appendix 1.

**Table 2:** Factors that clinicians consider when making decisions regarding telehealth.

Category and factor		Influence of factor
**Technology**		
	Client’s hardware	Telehealth is recommended if the screen size appropriate for the session type. SP^a^ sessions require a full-sized tablet or computer. Counseling services^b^ require only a phone.
	Client’s internet data plan	Telehealth (videoconferencing) is recommended only if sufficient internet data are available.
	Confidence with technology	Telehealth is recommended when the client, caregiver, and clinician are confident with technology.
	Functionality of telehealth platform	Telehealth is recommended if the platform had required features (eg, annotation feature for SP sessions).
	Telehealth-ready workspace	Telehealth is not recommended when the clinician had a shared office.
**Clients and families**		
	Client’s age	Telehealth for SP is more recommended for school-aged children than younger children, and telehealth for counseling is services is more recommended for adult clients than child clients.
	Client’s attention to screen	Telehealth is recommended when the client is perceived to have a sufficient attention span.
	Socioeconomic background	Telehealth is not recommended for clients from lower socioeconomic backgrounds.
	Caregiver’s management of child’s attention or behavior	Telehealth is not recommended for child clients whose caregivers were perceived to have difficulty managing their behavior.
	Barriers traveling to in-person therapy	Telehealth is recommended when barriers to traveling (eg, illness, transport, and work commitments) were present.
**Clinical services**		
	Type of clinical need	Telehealth for counseling services is recommended for parenting advice but not direct work with children; telehealth for SP is recommended for stuttering, early language, feeding, and school-aged language; and telehealth for OT^c^ is recommended for feeding.
	Presence of additional diagnoses or risk factors	Telehealth for counseling services is recommended for PND^d^ and DV^e^, and telehealth for SP and OT is recommended for clients without clinical comorbidities.
	Requirement for interpreter	Telehealth is not recommended when an interpreter is required.
	Assessment	Telehealth is not recommended for comprehensive initial assessment in SP and OT but is recommended for therapy.
**Clinician**		
	Access to “telehealth champion”	Telehealth is recommended when the clinician has access to a telehealth champion.
	Motivation for telehealth	Telehealth is recommended if the clinician is motivated to use telehealth.

^a^SP: speech-language pathology.

^b^Counseling services include those from a psychologist, counselor, or social worker.

^c^OT: occupational therapy.

^d^PND: postnatal depression.

^e^DV: domestic violence.

### Technology Considerations

The clinicians noted that appropriate technology hardware and internet data were required at both the client’s and clinician’s end for telehealth to be considered “doable” [counselor 3]. The hardware requirements for clients varied depending on the clinical service to be provided; speech-language pathology sessions typically required a laptop or full-sized tablet, while many counseling services required only a telephone, and occupational therapy or speech-language pathology sessions for pediatric feeding could be conducted with a smartphone (Table 2). In addition to having the required technology, clinicians were more likely to offer telehealth when the client or caregiver was confident with technology and willing to solve technological problems:

I think for me, trying to assess a parent’s technology literacy – their technical literacy using the internet, using their smart devices, being able to troubleshoot [is important when considering suitability for telehealth]. Trying to assess that first to see if this is doable is really important and part of the engagement period when we’re trying to figure out if telehealth is going to work.

Counselor 3

Clinicians were more likely to offer telehealth when they had ready access to clinical rooms that were telehealth enabled, felt confident about solving technological problems, and had telehealth-ready resources. The clinicians noted that the telehealth platforms available to them were slow and limited in interactive functionality, making telehealth not a viable option for some of the client’s goals:

In an ideal world, I probably wouldn’t choose to be using Pexip as our main platform because it limits the engagement with our clients. If we could use something along the lines of Zoom or something where we could give control over to the client, I think that would be really helpful, and we would be able to work on a wider range of goals.

SP8

### Client and Family Considerations

Clinicians considered factors related to the client such as the client’s age, attention, and concentration, the family’s socioeconomic advantage, and potential barriers to attending the community center. The counseling team considered adult clients to be more suitable for telehealth than children. Clinicians within the speech-language pathology team considered preschool and school-aged clients to be more suitable for telehealth than younger children. However, the client’s age interacted with the nature of the clinical work, such that an age that clinicians would normally consider less suitable for telehealth may be deemed appropriate due to the focus of the clinical work. This highlighted that clinicians often weigh up different, sometimes competing, factors in deciding to offer telehealth to specific clients as explained below:

I don’t want to disagree with myself, but I said preschool to school age [clients were most suitable for telehealth], but then I’m thinking “actually I’ve had so much success with you under threes as well”...A section of those under threes are also really suitable for telehealth because the parent training approach works really well with telehealth.

SP1

Clinicians considered a client’s ability to attend to a screen, and those who could attend to a screen were deemed more appropriate for telehealth. Clinicians also considered the family's socioeconomic status; families with low socioeconomic advantage were considered less likely to be suitable for telehealth due to the cost of internet data and hardware. Potential barriers to in-person attendance were also considered, and if illness, number and age of children, time of session, and location of employment would make attendance difficult, telehealth sessions were more likely to be recommended.

### Clinical Service Considerations

Clinical factors such as the client’s diagnosis, the type of intervention, the requirement for an interpreter, and whether the session was an assessment or therapy session were considered when making a recommendation about telehealth. In general, assessments were considered less suitable for telehealth, as were sessions requiring an interpreter. Each profession considered some areas of their scope to be more suited to telehealth than others. For example, clinicians in occupational therapy considered telehealth to be suitable mostly for treatment and pediatric feeding challenges rather than fine or gross motor issues. Clinicians in speech-language pathology considered telehealth to be suitable for stuttering, feeding, and language clients but not for speech sound disorders, and counseling clinicians considered telehealth to be suitable for one-on-one sessions (eg, providing parenting strategies and one-on-one counseling) but not for group sessions or family counseling.

Table 2 contains more details and example quotes are provided in Multimedia Appendix 1.

### Clinician-Related Considerations

Clinicians were more likely to offer telehealth if their peers were positive about telehealth, used it regularly, and were willing to share their experiences. Many clinicians referred to these people as “telehealth champions” [OT2]. Speech-language pathology clinicians had a greater uptake of telehealth during the pandemic and sustained use than clinicians from counseling or occupational therapy. Counselor 3 considered this to be attributable to the number of telehealth champions across the disciplines, as explained below:

Speech pathology have adopted [telehealth] really well. I think there was some more champions for telehealth within their team who were more keen...whereas for counselling, I don’t think we, in our team, had as many champions. I think having more people who are interested, passionate about it in my team would have made a difference.

Counselor 3

Compared with in-person sessions, telehealth sessions were perceived to require more preparation time and the development of new skills and to be more tiring, as explained below:

I’m using a lot of telehealth, but I found it’s more tiring, and it draws on a lot more energy, because I think you’re concentrating and things like that. I do think it’s important for the clinicians to look after ourselves, to be aware of our limits, our resources, and the support.

Counselor 1

Clinician confidence was a key factor in them recommending telehealth for their clients. The more they used telehealth and considered it to be part of their routine practice, the more their confidence grew, and the more they recommended it to their clients, setting up a positive feedback loop. Conversely, as noted by counselor 3, “If you’re not confident, you hardly use it; it’s like you’ll literally forget about telehealth.” Clinicians reported weighing up competing factors when considering whether to recommend telehealth. Even when they considered telehealth to be clinically appropriate for their client, they weighed this factor against their own motivation and available resources to provide telehealth. This tension between competing factors was explained by counselor 1: “It’s important to provide a good service but at the same time we do need to balance out the impact on the clinicians. It’s a balancing act.”

In response to our second question regarding the clinicians’ perceptions of telehealth utility outside of COVID-19 lockdowns, the clinicians conveyed 3 themes. These were “flexible telehealth use,” “telehealth can be superior to in-person therapy,” and “fear that in-person services may be replaced.”

### Flexible Telehealth Use

The clinicians explained that their use of telehealth was not “all or nothing” and that they used telehealth when it was appropriate for a specific purpose with a given client. Like all clinical tools, the clinicians felt that telehealth was best used in specific circumstances, as explained below:

I feel confident in being able to say yes, I can offer – like for this particular client, I can offer a good service via telehealth. Whereas for others I also feel confident in saying I don’t think telehealth would be a good fit for this client, their goals, and just their overall situation.

SP6

For some clients, the clinicians used a hybrid service delivery, moving between telehealth and in-person sessions within 1 treatment block. For other clients, the clinicians started with telehealth and moved to (and remained providing care) in-person once they had built engagement and trust with their client.

People start with a phone call, with a telehealth session and then, “can I come and see you face-to-face?” It just–it evolves...In my perspective I find telehealth’s benefit is very much in building the engagement and the trust in people.

Counselor 2

At other times, clinicians started a client’s service block in person and finished it using telehealth. For example, how they use telehealth at the end of an in-person speech-language pathology treatment block to increase the client’s generalization of skills and the parent’s responsibility for their child’s care is explained below:

I start them off on face-to-face and then, towards the end of the block, transition them to telehealth to encourage the parent to take on board the fact that these sessions aren’t going to go forever and help them to learn how to help their child.

SP3

Clinicians also used telehealth flexibly to enable attendance at a primarily in-person service when illness or transport difficulties would have prevented a client from attending the clinic.

### Telehealth Can be Superior to In-Person Therapy

The clinicians described how, in some circumstances, telehealth enabled them to deliver better care than in-person service. Via telehealth, clinicians can see clients in their home environment, enabling observations of naturalistic parent-caregiver interaction, children’s communication, and infant feeding context and behavior. Telehealth enables clinicians to include both parents in their children's counseling care, and how this is helpful for some fathers is explained below:

We try to have mum and dads [sic] as part of therapy. Having just the mum is not helpful if it’s a family with a mum and a dad. So, I can see lots of potential in telehealth, in having a dad who can get out during his lunchbreak for example and join in on the session with us. Telehealth gives us all these options.

Counselor 3

Clinicians considered telehealth to be richer than in-person care when they reimagined and adapted their clinical role, instead of replicating an in-person session. For example, the counseling team felt that, when they adapted their in-person child-directed counseling sessions to be telehealth parent-coaching sessions, the intervention was more effective. Counselor 3 described this reconceptualization of the session as resulting in “less direct contact with the child, but probably more meaningful intervention” that is then able to be delivered by the parent “160 something hours a week rather than only one hour a week [by the clinician].” Similarly, speech-language pathology clinicians found that adapting their direct delivery of therapy to become telehealth parent coaching increased parent confidence, empowerment, and engagement, as explained below:

In those instances where I wanted the parent to be more involved or I wanted the parent to have the confidence to help their child, I was using telehealth as an opportunity to be explicit about doing some coaching and giving the parent the power, saying, “look, I can’t hear and see as well as you can, so why don’t you have a go. You give the child some feedback and I can coach you through what the next step is.”

SP5

Occupational therapy clinicians, however, described feeling that occupational therapy sessions were not suitable for telehealth because in-person sessions could not be replicated by parents at home via telehealth. This concern about parents being unable to replicate an in-person occupational therapy session was explained by OT1, “In an OT session we set up lots of things on the table. We do a variety of tasks which is not possible for parents to organise at home.” The senior OT considered that the limited use of telehealth by the OT team was related to the inability of the OT team to identify ways of adapting sessions for telehealth, as explained below:

So, I guess, for the OT team, they really needed a chance to discuss and say, “well how would we work on this skill at home when they don’t have access to specific things? How could we use household items?”...Like if there was a parent there, how could we then coach the parent to then do some hands-on assistance to then help the child.

OT2

So, when considering telehealth, clinicians also considered how easily their in-person services could be “translated” to the synchronous online environment. As in this example from occupational therapy, when this “translation” from offline to online delivery of care was considered too difficult (eg, because of limited relevant equipment on the client side), telehealth was considered less appropriate.

### Fear That In-Person Services May be Replaced

Clinicians expressed fear that telehealth may, in the future, fully replace in-person services. Despite the benefits outlined above, clinicians shared that there were elements of their role that they felt could not be replicated in telehealth modality, such as observations of body language with counseling clients, hands-on activities for occupational therapy clients, and therapy for speech sounds, as noted below:

I’m scared that [telehealth] is going to replace the interpersonal face-to-face at one stage. That’s what concerns me because I don’t think anything would replace the face-to-face...When it comes to a relational thing that you’re doing, you need to notice other stuff and the little bits which you cannot notice if it’s not face-to-face.

Counselor 2

The clinicians felt that many clients preferred in-person sessions, and in some cases, the clinicians, themselves, preferred in-person services. The preferences of clients and clinicians are explained below:

Our families aren't valuing telehealth as much as they value sessions in-person. Even when I felt that I was offering a very similar service, I felt like I really had to convince the family that it was the same quality, and that we were working towards the same goals, and it was still an effective service, even if delivered via telehealth.

SP8

I personally prefer face-to-face in everything but there are advantages of the telehealth.

Counselor 2

Given their belief that there are elements of clinical service that cannot be fully replaced by telehealth, the clinicians felt the current hybrid option better suited their needs than a telehealth-only service provision.

## Discussion

### Overview

The current investigation sought to explore the experiences of allied health clinicians working within a community-based health service during a period of rapid transition toward telehealth. Of particular interest were the factors that informed clinicians’ decisions regarding the suitability of telehealth services for individual clients and their families. The second area of exploration was clinician perceptions about the use of telehealth beyond the context of public health–driven lockdowns due to COVID-19. A dominant theme throughout this investigation was the complexity and multifaceted nature of clinical decision-making with both individual and organizational factors playing the greatest role in supporting this decision-making.

### Principal Findings

The findings of the current investigation reinforce the notion that while some general principles underpin clinicians’ telehealth recommendations, clinical decision-making is client focused, complex, and dynamic [43,44]. Clinicians considered the technology available, client factors, their own personal experiences and preferences, and clinical resources alongside considerations regarding the service to be provided. The decision as to the suitability of different families for telehealth services was not static. As with all clinical decisions, the decision about the suitability for telehealth was embedded in an understanding of what was best for any client at any one point in time. It was not as simple as identifying that older children were more suitable than younger children—in fact, younger children were sometimes more suitable than older ones—or that one intervention approach was more easily adapted for telehealth than another.

The clinicians readily acknowledged that their own personal factors and preferences also informed their decisions. Clinician confidence in providing telehealth services, and feeling supported to do so, was a key driving factor for telehealth use among the participants interviewed. The positive feedback loop reported by the clinicians was consistent with earlier investigations where prior experience, success, and confidence informed future decision-making and practice [43].

### Clinical Decision-Making

The clinicians in the current investigation described decision-making that was consistent with both the information-processing approach and intuitive-humanist approaches [21]. Most clinicians described a process of systematically considering technology, client and family factors, the clinical service required, and their own resources in an information-processing model. Clinicians who had more experience with telehealth reported practices that aligned with an intuitive-humanist approach, as their experience allowed them to recognize patterns of factors that were associated with telehealth success. This study is among the first to describe the factors that allied health clinicians consider when deciding on a client’s suitability for telehealth. The awareness of these factors will help guide clinicians newer to telehealth, as it will provide a reference point of things to consider when using an information-processing model for telehealth decisions.

### Clinical Decision-Making and E3BP

Many of the decisions described by the participants of this investigation were embedded in a desire to improve client outcomes using the evidence available to them. However, there was a disconnect between some of their perceptions and research literature regarding the effectiveness of telehealth for specific clinical groups, tasks, and disorders. Although research indicates that telehealth is appropriate for allied health assessments [2], children with additional diagnoses or developmental concerns [45], the treatment of speech sound disorders [46], and occupational therapy [47], the clinicians’ internal evidence was that telehealth had not been successful in these areas and, therefore, should not be recommended with future clients. It is possible that the diverse socioeconomic and linguistic background [48] of the health district differs from the context of the external studies, and that this explains the conflict between the 2 sources of evidence. However, discrepancy between the external evidence base and clinician’s perceptions was also noted in a study of hospital-based allied health professionals representing a wider geographical area [15]. The authors noted that even when there were clear benefits of telehealth, patients were not always given the choice, and many decisions were “overridden by what is easy and efficient for the clinician” [p.3]. Within our study, though, participants described negotiating their own preferences, client factors, and clinical resources, rather than simply prioritizing their own comfort levels. It is also possible that other factors contributed to our clinicians’ beliefs about the effectiveness of telehealth, such as informal collegial discussions and the presence (or absence) of “telehealth champions.” Further exploration of the relative weight given to different parts of E_3_BP when making telehealth decisions is warranted, particularly the contribution of the third element of E_3_BP, the preference of an informed client or caregiver.

The participants in this study indicated that telehealth should be available as a service delivery option but should not fully replace in-person services. Indeed, the option to use telehealth after a thorough consideration of client and clinical factors is recommended by many allied health professional associations [19]. Given the reduction in telehealth use when pandemic public health restrictions were eased [49], it is helpful to consider the clinicians’ decision-making within an implementation science model such as the NASSS framework [50].

### Factors Impacting the Ongoing Implementation of Telehealth

Embedded within the clinical decisions of client suitability were a number of factors that could inform the ongoing implementation and sustainability of telehealth in community-based services. Consistent with the NASSS framework [50], the clinicians specifically considered factors related to condition, technology, adopters, and organization when considering client suitability for telehealth. Specifically, different clinician groups considered distinct factors related to client diagnoses and comorbidities when determining who would not be suitable for telehealth.

With regard to technology factors, the clinicians noted that the use of Pexip was, in some cases, a barrier to participation in telehealth when other tools such as Zoom were more widely used and had greater functionality. The use of a less familiar videoconferencing platform with limited end-to-end functionality may increase the complexity of the technology factors when considering client suitability and reduce the likelihood of sustainable implementation. Namely, technologies that are less familiar and require detailed instructions or support increase the complexity of the implementation [13,50]. Furthermore, if clinicians cannot mirror the work of in-person tasks in a web-based format (eg, through screen sharing or screen annotation), the technology will be a factor in complicating the implementation.

The adopter system within the NASSS model is the domain that placed the greatest burden on participating clinicians. The clinicians reported increased complexity in the tasks and environment required for telehealth (eg, availability of telehealth-suitable resources and shared workspaces); this was predominantly due to the relative novelty of telehealth service provision before the global pandemic. Across each of these domains, an increase in complexity reduces the likelihood of sustained implementation [50]. That said, some areas of practice were identified as being optimally suited to telehealth service provision (ie, communication support for the families of children under 3), thus, reducing the complexity of adopting the new model of care. Finally, organizational factors were reported to drive the implementation.

The circumstances of the shift to telehealth fostered a high-drive environment for change and the emergence of “telehealth champions” meant the shift to telehealth was less pronounced in some areas of clinical practice than others. For example, the work of an OT during a feeding evaluation or therapeutic intervention requires less adaptation for a telehealth context than the work required for the adaptation of fine and gross motor therapies. Organizational factors that complicated the provision of telehealth included the perception of an increased workload, limited shared knowledge across client care, and the group “work” of making the transition.

### Limitations

This was a small study, conducted at 1 child and family health service, at a specific point between COVID-19 outbreaks in Australia. Further confirmation of the findings in larger studies in other geographical areas would be beneficial. Our participants only represented 5 of the allied health professions. Although we used decision-making frameworks to describe our results, our study design did not specifically explore the mechanism the participants used when making decisions.

### Conclusions

Recent world events provided an opportunity for telehealth uptake within systems that had not previously embraced it as a method of service delivery. This project asked clinicians to reflect on their clinical decisions regarding client or family suitability for telehealth and their perceptions of telehealth use beyond the pandemic period. Understanding these perspectives, occurring within a service that had limited previous uptake of telehealth, highlighted the different factors that clinicians consider. As a part of this complex decision-making, clinicians balanced the individual clinical needs of the client alongside themselves as a clinician, the organizational context of their service, and the wider societal benefit of tele-provided services.

## Appendix

Multimedia Appendix 1 Example quotes of factors that clinicians consider when making decisions regarding telehealth.

## Abbreviations

CFAHS: Child and Family Allied Health Services
CNSLR: counselor
EBP: evidence-based practice
NASSS: Nonadoption, Abandonment, Scale-up, Spread, and Sustainability
NSW: New South Wales
OT: occupational therapist
SP: speech-language pathologist

## Data Availability

The data sets generated during this study are available from the corresponding author on reasonable request.
