# Supplementary material for: Clinicians’ Decision-Making Regarding Telehealth Services: Focus Group Study in Pediatric Allied Health
Source: JMIR Form Res. 2024 Jun 7;8:e46300. doi: 10.2196/46300 (PMC11193077; doi:10.2196/46300)
Supplement: Multimedia Appendix 1 [file formative_v8i1e46300_app1.docx]

**Table S1.** Example quotes of factors that clinicians consider when making decisions regarding telehealth

| **Category** | **Factor** | | **Influence of factor on decision making** | **Example quotes** |
| --- | --- | --- | --- | --- |
| Technology | Client hardware (computer, tablet etc) | | Screen size appropriate for session type. SP prefer full-sized tablet or computer; adult CNSLR sessions require only a phone | SP3: They have got at least a laptop or an iPad, for the purpose of the screen size, and the internet connection is good.  SP1: With breast feeding clients, phone is great because there’s huge capacity to move around depending on the view or the perspective they need to show.  CNSLR4: I’ve had a lot of my clients really effectively do their entire 16 sessions of counselling over smart phones...but I’m doing three to four hours of sessions a day and I can’t do that on the smart phone because I’m the one doing the presentations and craning my neck. |
|  | Client’s internet data plan | | Client needs sufficient data plan for videoconferencing | SP10: A lot of our families in [suburb with low socioeconomic advantage] didn’t have access to the technology, didn’t have access to the internet and the data.  SP7: My families have similar issues to SP8 -things like insufficient technology or devices. Maybe even being on credit plans for phones and internet, where they don’t have insufficient credit or they can’t afford to use that much on a session, so they decline telehealth for that reason. |
|  | Confidence with technology (client/ carer and clinician) | | TH recommended more frequently when client, carer and/ or clinician are confident | CNSLR3: [It’s important to] assess a parent’s technology literacy – their technical literacy using the internet, using their smart devices, being able to troubleshoot. Assessing that first to see if this is doable is really important.  SP4: When telehealth has worked that gives me the confidence that I can try and extend and expand this to other client groups. Yeah, but when things were hard I did not have the confidence to say ‘yeah, let’s do it’. |
|  | Functionality of telehealth platform | | TH features (e.g. annotation) required for SP client sessions | SP3: We’ve been using Pexip mostly for our telehealth and it’s not interactive. I know there are other platforms where the client can do things, but really from my end I’m the one moving the stamps or moving the bits around the screen. I’ve had kids say, ‘Can I do it? Can I do it?’ I’m like, ‘No, sorry, it doesn’t work like that’. So if I could wave a magic wand, I’d use a different technology where the client could have some interaction. |
|  | Clinician has telehealth-appropriate workspace | | Shared office or requirement to pre-book a TH-enabled space decreases TH use | OT2: The decision about whether to use telehealth comes down to the clinicians’ confidence with using the technology and troubleshooting.  SP3: I see telehealth clients in the office where I sit whereas SP5 sits in a shared office. To do telehealth she has to book a different room.  SP5 ... and I have to set up, and that other computer doesn’t load everything how mine does and… that’s 15 minutes extra prep you don’t need. |
| Client and family | | Age | SP – TH recommended for school-aged compared to younger children, except for feeding and early language; Couns. - Adult clients recommended for TH more than child clients | SP6 With school-age clients, I’d be more likely to offer telehealth or trial it because the attention is often there a little bit more and they’re a bit more familiar with the devices.  OT2: What else do we consider? I guess the age of the client.  CNSLR3: I don’t think I’ve really done much direct engagement with children using telehealth. Individual adults for individual counselling, it works really well. |
|  | | Ability to attend to screen | Clients require sufficient attention span for TH to be recommended | OT1: If the child is having attention issues, it’s really hard for them to sit and stare at the screen…it’s hard for them to engage in telehealth if they have attention issues.  CNSLR4 [it was successful because] it was and adult who I was working with. His attention span was long enough to maintain focus virtually |
|  | | Socio-economic factors | Clients from lower socio-economic backgrounds less likely be recommended for TH | CNSLR3: Some of the families are quite financially disadvantaged, so I check to see how they would access the internet if they’re going to use their own internet... Especially if I knew a family are financially vulnerable then we may consider not using telehealth because it’s going to be too expensive. |
|  | | Carer’s capacity to manage child’s attention and behaviour | Child clients whose carers were perceived to have difficulty managing their behaviour were less likely to be recommended for TH | SP6: [Talking about what makes telehealth difficult] Where the parent perhaps is not as prepared to manage behaviours and manage the environment, manage the setup, motivate the child, yeah, have an awareness of when the child’s needing the cues. |
|  | | Barriers travelling to in-person therapy | Barriers to travelling (illness, transport, work commitments, young baby) increased likelihood of TH recommendation | SP4: [I offer telehealth] if there are transport issues, the sibling is unwell, or if they have to isolate with COVID restrictions, if they’ve got cold and flu symptoms, if the session is close to school pick up and drop off time.  CNSLR1: A lot of my clients are new parents. I think the age of the babies and also the number of young children are factors I will consider... Also, sometimes if they have young children like a newborn baby and a toddler, or a couple of other young children it’s very difficult for them to travel, even if they’ve got their own transport. |
|  | | Diagnosis / risk factor | Couns: Domestic Violence – location of the violent partner influenced decision about TH; Post Natal Depression – more likely to recommend TH  SP and OT – Clients with clinical comorbidities less likely to be recommended for TH | CNSL3: One of the things we need to assess is if there are high conflict relationships at home or domestic violence risks. For those families where there is coercive controlling behaviours, we find it challenging for telehealth sessions to happen because there would be no space that would be appropriate in the home setting.  CNSLR4: If there’s been some domestic violence issues flagged, then I will not consider telehealth, I pretty strongly insist on people coming in face to face.  SP1: I think the ones who were showing a lot of autism flags, or other sort of developmental issues was just not as effective [by telehealth]  SP6: [Telehealth is more difficult when there are] those concomitant issues, child wise; if they have got attention deficits or any kind of hearing concern or challenging behaviours.  SP1: A client that does not have additional diagnosis is a good [telehealth] candidate, in a sense. |
| Clinical services | | Clinical work | Couns. – TH recommended for parenting advice, but not for direct work with child clients  SP – Less likely to recommend TH for speech sound disorders, More likely to recommend for stuttering, early language groups, feeding, school aged language  OT- TH recommended for feeding, but not for other areas | CNSLR4: I found that focussing just on a parent-based strategy over telehealth is much more effective...compared to interaction-based therapies  CNSLR4: One of the things that I’ve learnt to really consider is if the presenting issue usually requires me to work with the child directly, and if it’s a child or adolescent, my very strong preference is face to face. If the presenting issue is likely to be mainly parenting work, or individual adult counselling, telehealth is probably what I’ll be offering first.  CNSLR2: [Telehealth is good for] information exchange... but when it comes to a relational thing that you’re doing, you need to notice other stuff and the little bits which you cannot notice if it’s not face-to-face.  SP8: In speech pathology, there were a number of clinical areas where it’s just not effective to offer the service via telehealth...So, for example, I felt quite hesitant to target speech sound disorders via telehealth because I couldn’t be certain that I was hearing exactly what the child was saying because of poor audio quality. I wasn’t also able to rely so much on the child’s facial cues and whatnot because the visual clarity wasn’t always there.  SP5: I mentioned stuttering and language [as being good for telehealth, and] I think feeding’s another one that lends itself to telehealth because you can see the family, the set up at their home. If they come into the clinic, it’s very artificial, whereas you get a window into where the child sits, what the room looks like, where the TV is, what implements, bowls, spoons whatever they’re using at home.    OT1: It’s not going to be easy for a child with sensory needs to be sitting in front of the screen. |
|  |  | Need to use interpreter | TH less likely when interpreter was required | SP1: If the family requires the use of interpreters, it’s another factor. We’re really fortunate to be able to offer video conferencing telehealth interpreting services which is great, but I found so many issues coming up between having to balance my screen, interpreter’s screen, parent’s screen and then naturally this idea of the flow of conversation is disrupted |
|  |  | Assessment vs therapy | Full assessments preferred in-person, however screening assessments trialled via TH by SP and Couns. | SP2: Doing an assessment face to face is much easier and on telehealth it’s much harder, especially formalised assessment - the results are probably not even valid  SP7: Other times I found [telehealth] going well for me was with screening appointments and new referrals where, typically, we’d bring them in for a half an hour or 45-minute sessions just to meet them. That could be done relatively easily in a Zoom session – Pexip session.  CNSLR3: So we do [our intake screening sessions] sometimes on telehealth too, a small percentage. So yeah, so some initial engagement sessions are by telehealth. But currently the majority is still face-to-face.  OT1: But it is very difficult because all of our OT assessments do require that we see them perform particular skills in certain timeframes and ...being able to have parents be able to use the cameras well enough to show us whole body or close-up of the hand or that kind of thing is challenging. So it is a bit trickier to do assessments |
| Clinician | | Access to ‘telehealth champions’ | Access to telehealth ‘champion’ increased likelihood of TH recommendation | CNSLR3: I think if you get enough people, it makes learning easier. People can share strategies, faster ways of connecting, which platform works better, how to do this online. I think it’s just more learning and cross-promotion between clinicians that way.  OT2: We had a smaller team and it did take longer for people to get confident and comfortable |
|  |  | Motivation and energy for learning something new | Clinician required sufficient motivation and energy to recommend TH | SP4: So, you still have to work on your own skills. It’s not just the family, it’s how much work I put in to get myself prepared. With face-to-face sessions, you just grab toys and you start doing it whereas with telehealth you have to have things ready on the screen to get going with that.  OT2: The clinicians are so much more familiar with things face-to-face. It’s out of their comfort zone...for the clinicians to do telehealth. Apart from the champions who, I guess, really embraced it and really got on board with it. I think for majority of clinicians, it’s a bit more work and a bit more effort to do the telehealth.  SP5: I just personally prefer face-to-face. I suppose it depends on your caseload at the time as well. If you’ve got a little – a whole bunch of kids with challenging behaviours and it’s hard enough even face-to-face to get those interactions and communication going |

SP = Speech-Language Pathologist; OT = Occupational Therapist; CNSLR = Counsellor; TH = telehealth; S = senior; Pexip = video conferencing platform (https://www.pexip.com/)
